# Graft-versus-Host Disease Is Enhanced by Selective CD73 Blockade in Mice

**DOI:** 10.1371/journal.pone.0058397

**Published:** 2013-03-08

**Authors:** Long Wang, Jie Fan, Siqi Chen, Yi Zhang, Tyler J. Curiel, Bin Zhang

**Affiliations:** 1 Cancer Therapy & Research Center, Department of Medicine, University of Texas Health Science Center, San Antonio, Texas, United States of America; 2 Robert H. Lurie Comprehensive Cancer Center, Department of Medicine- Division of Hematology/Oncology, Northwestern University Feinberg School of Medicine, Chicago, Illinois, United States of America; 3 Department of Bioengineering, Center for Cell Biotherapy of the First Affiliated Hospital, Zhengzhou University, Zhengzhou, Henan Province, China; University of Colorado Denver, United States of America

## Abstract

CD73 functions as an ecto-5′-nucleotidase to produce extracellular adenosine that has anti-inflammatory and immunosuppressive activity. We here demonstrate that CD73 helps control graft-versus-host disease (GVHD) in mouse models. Survival of wild-type (WT) recipients of either allogeneic donor naïve CD73 knock-out (KO) or WT T cells was similar suggesting that donor naïve T cell CD73 did not contribute to GVHD. By contrast, donor CD73 KO CD4^+^CD25^+^ regulatory T cells (Treg) had significantly impaired ability to mitigate GVHD mortality compared to WT Treg, suggesting that CD73 on Treg is critical for GVHD protection. However, compared to donor CD73, recipient CD73 is more effective in limiting GVHD. Pharmacological blockade of A2A receptor exacerbated GVHD in WT recipients, but not in CD73 KO recipients, suggesting that A2 receptor signaling is primarily implicated in CD73-mediated GVHD protection. Moreover, pharmacological blockade of CD73 enzymatic activity induced stronger alloreactive T cell activity, worsened GVHD and enhanced the graft-versus-leukemia (GVL) effect. These findings suggest that both donor and recipient CD73 protects against GVHD but also limits GVL effects. Thus, either enhancing or blocking CD73 activity has great potential clinical application in allogeneic bone marrow transplants.

## Introduction

Acute graft-versus-host disease (GVHD) is a primary T-cell-mediated complication associated with allogeneic hematopoietic stem cell transplantation, leading to high post-transplant morbidity and mortality [1]–[3]. Alloreactive donor T cells recognize disparate histocompatibility antigens of the recipient and cause progressive damage to target organs such as skin, liver, and the gastrointestinal tract. Proinflammatory cytokines enhance the generation of donor anti-host cytotoxic function [4], [5]. Current therapies for acute GVHD are limited and mortality remains high despite treatments [1]–[3]. Thus, strategies to control GVHD development by altering the proinflammatory environment or the cellular effectors that are critical in mediating acute GVHD could be highly effective.

CD73, known as ecto-5′-nucleotidase (ecto-5′-NT, EC 3.1.3.5) [6], [7], sequentially phosphohydrolyzes adenine nucleotides, leading to adenosine generation in tandem with CD39 (ecto-ATPase) [8]. In particular, CD73 hydrolyzes the phosphate group from AMP to generate adenosine. Recent *in vivo* studies implicating CD73 in a variety of tissue protective mechanisms have provided new and important insight into its regulation and function [6], [7]. A number of studies have suggested that CD73-generated adenosine plays a crucial role in many processes including leukocyte extravasation [9], [10], cellular immunoregulation [6], [11]–[13] and cardioprotection [14]. Modulation of inflammation by CD73-mediated adenosinergic signaling via specific adenosine receptor subtypes has been characterized in various murine models, including T cell–dependent autoimmune encephalomyelitis [15], colitis [16], [17], infections [18], and in anti-tumor T cell immunity [19]–[23]. The thromboregulatory effects of CD39 have been reported in cardiac transplantation models [24], [25]. Moreover, GVHD could be enhanced by extracellular ATP as a danger signal [26]. However, very little is known about CD73 as an effector arm of the immune or inflammatory response in acute GVHD. Interestingly, there are clear demonstrations of the importance of the CD73/adenosine axis in murine skin [11], cardiac [27] and lung [28] transplantation models.

Given that CD73 is closely involved in multiple processes vital to successful transplantation such as vascular permeability [29], leukocyte adhesion [30], [31], ischemic preconditioning [14], [32] and immunosuppression [11]–[13], and is important in solid organ allograft rejection [11], [27], [28], we hypothesized that CD73 would play a critical role in the T cell-mediated development of acute GVHD. In the present study, we analyzed the ability of WT and CD73 KO donor T cells to proliferate, produce cytokines, infiltrate host issues and cause systemic acute GVHD. We further examined the contribution of recipient CD73 and A2A adenosine receptor (A2AR) to GVHD. Finally, the effects of pharmacological blockade of CD73 using the selective inhibitor α,β-methylene adenosine 5′-diphosphate (APCP) in both GVHD and GVL models were assessed.

## Results

### CD73 Affects GVHD Development

**Figure 1:**
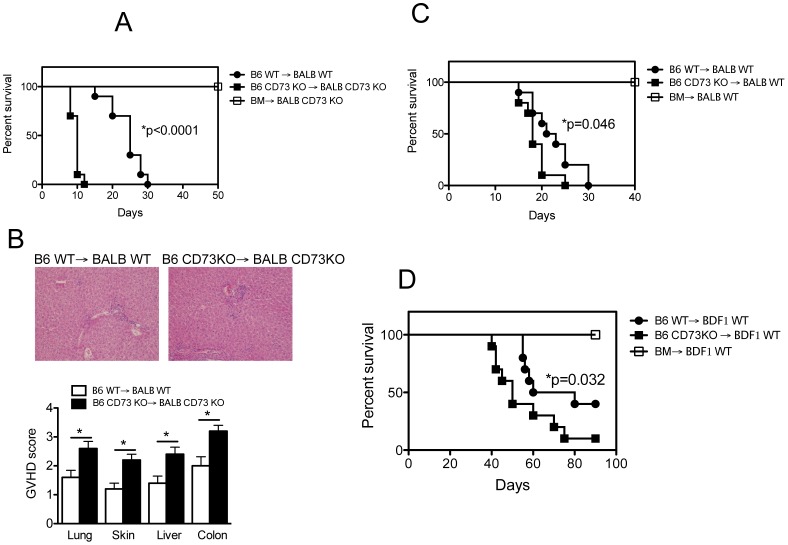
CD73 affects GVHD development. Lethally irradiated BALB/c CD73 KO (**A**) and WT **(C**) or B6D2F1 (BDF1) (**D**) mice were given i.v. injections of 5×10^6^ T cell depleted bone marrow (BM) cells from C57BL/6 (B6) mice donors alone (n = 5) or with 2×10^7^ splenocytes from WT (n = 10) or CD73 KO (n = 10) B6 donors. P values indicate the differences between recipients receiving WT versus CD73 KO cells. Results are representative of 2 independently performed experiments with similar results. (**B**) Ten days later, pathological analyses of lung, liver, skin, and colon of recipient mice (**A**) receiving WT or CD73 KO SP were performed by H&E staining. Combined results of pathology scores for 5 mice in each group. Error bars indicate the standard errors of the mean in the group. Representative micrographs from recipient livers (magnification 200X) are shown.

Initially we asked whether CD73 plays a role in the development of GVHD. To this end, we collected total splenocytes from either WT or CD73 KO B6 mice and transferred them together with T cell–depleted (TCD) bone marrow (BM) cells into lethally irradiated WT or CD73 KO BABL/c recipients. As expected, mice receiving TCD BM cells alone had no sign of GVHD and all survived. Interestingly, typical clinical features of GVHD, including hunched back, ruffled fur, hair loss, diarrhea, and body weight loss (data not shown) were observed in recipients of either WT or CD73 KO splenocytes. WT splenocytes recipients all died within 30 days of severe GVHD (Figure 1A). By contrast, transfer of CD73 KO splenocytes into CD73 KO recipients dramatically shortened recipient survival and elicited profound weight loss following acute collapse (Figure 1A). Consistent with the shortened survival, enhanced clinical severity of GVHD was observed in the absence of CD73 (Fig. 1B). We next asked whether donor CD73 plays a role in the development of GVHD. Compared with CD73 KO recipient mice receiving allogeneic CD73 KO cells (Figure 1A), WT recipient mice receiving allogeneic CD73 KO cells survived longer, but died earlier than WT recipient mice receiving allogeneic WT cells (Figure 1C), suggesting a role of donor CD73 in GVHD. In the second model, parent-to-F1 transfer was employed to generate severe GVHD by lethally irradiating B6D2F1 mice and transferring TCD B6 BM cells plus either WT or CD73 KO B6 splenocytes. Mice receiving WT splenocytes developed GVHD, and about 40% of them survived beyond 80 days, whereas less than 10% of mice receiving CD73 KO splenocytes survived that long (Figure 1D). Together, we conclude that CD73 is implicated in GVHD development, and findings using fully major histocompatibility complex (MHC)-mismatched BM transfer and parent-to-F1 transfer models indicate a specific role of donor CD73 in GVHD pathogenesis.

### CD73 is Required by Donor CD4^+^CD25^+^ Tregs, Rather than Naïve T Cells to Limit GVHD

**Figure 2:**
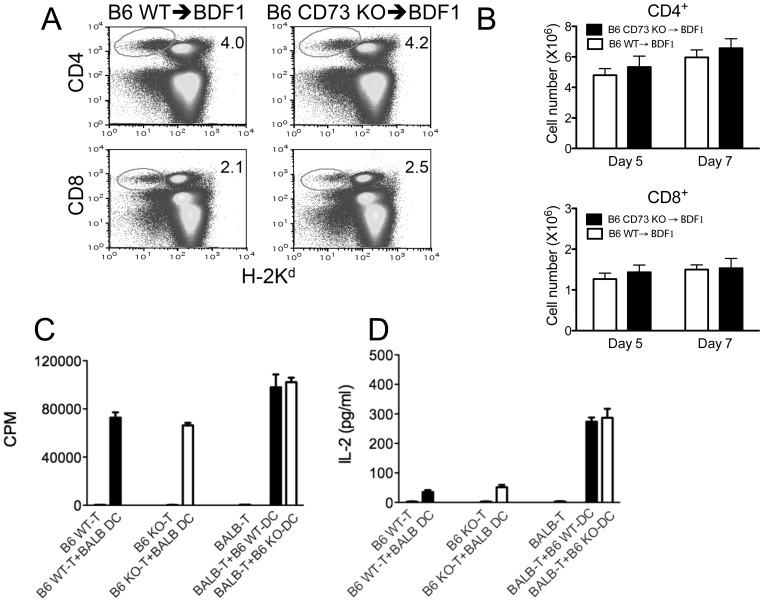
Naïve CD73 deficient (KO) T cells exhibit normal activation and proliferation in response to alloantigen. B6 naïve WT or CD73 KO spleen T cells (CD25^−^CD62L^+^) (5×10^6^) were injected i.v. into B6D2F1 (BDF1) mice. After 5 days, recipient spleens were harvested and stained with anti-H-2K^d^ antibody together with either anti-CD4 or anti-CD8 antibodies. (**A**) Percentage of donor (H-2K^d^ negative) CD4^+^ or CD8^+^ T cells in total spleen cells was determined by flow cytometry. Numbers in quadrants indicate percent positive cells in each. Absolute numbers of donor T cells in recipient spleen (n = 4) (**B**) and liver (n = 4) (**C**) were determined at 5 and 7 days after T cell transfer. (**D**) B6 WT or CD73 KO T cells (5 × 10^5^/well) were co-cultured with 1 × 10^5^ BALB/c mouse CD11c^+^ dendritic cells (DC) for 3 days. To determine the role of CD73 on DC, BALB/c WT T cells (5 × 10^5^/well) were co-cultured with 1 × 10^5^ B6 WT or CD73 KO CD11c^+^ DC for 3 days. Alloreactive T cell proliferation was measured by [^3^H]thymidine incorporation. CPM, counts per minute. (**E**) Culture supernatants were collected for IL-2 detection by ELISA. Data are given as means ± SEM. Results are representative of 2 independently performed experiments with similar results.

Previous work demonstrated that memory T cells fail to induce GVHD [33]. Thus, it is possible that the enhanced ability of CD73 KO splenocytes to induce GVHD was due to increased number and/or activity of naive T cells. To test this possibility, we examined the proportion of memory T cells in donor spleen cells by flow cytometry. Frequencies of CD44^high^CD62L^low^ (effector memory) and CD44^high^CD62L^high^ (central memory) cells among splenic CD4^+^ or CD8^+^ T cells from WT mice were equivalent to those from CD73 KO donor mice (Figure S1). We further purified CD25^−^CD62L^high^ naïve T cells from WT or CD73 KO donor mice. To monitor the fate of donor naïve T cells during GVHD development, purified B6 WT or CD73 KO CD25^−^CD62L^high^ naïve T cells were transferred into BDF1 recipients i.v. The prevalence and absolute number of CD73 KO donor CD4^+^ or CD8^+^ T cells in recipient spleens were identical to those of WT donor CD4^+^ or CD8^+^ T cells 5 and 7 days after T cell transfer (Figure 2A, B). Similar donor WT or CD73 KO T cell accumulation was also observed in recipient liver lymphocytes (Figure 2C), suggesting that the differential GVHD susceptibility is likely not due to differing cellular distribution. We also found that CD73 deficiency had no effect on Th1, Th2 or Th17 commitment by intracellular cytokine staining of IFN-γ, IL-4 and IL-17 gating on donor type T cells (Figure S2). We next asked whether CD73 deficiency affects naïve T cells activation or proliferation in response to alloantigen *in vitro*. In an allogeneic mixed lymphocyte reaction **(**MLR), there was no difference in proliferation (Figure 2D) or IL-2 production (Figure 2E) between WT and CD73 KO T cells when co-cultured with allogeneic dendritic cells (DCs). Moreover, alloreactive T cell responses induced by WT or CD73 deficient DCs were comparable (Figure 2D, E).

**Figure 3:**
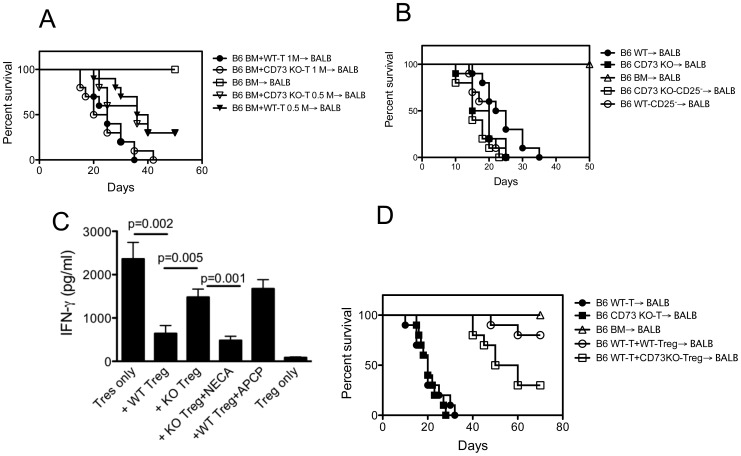
CD73 deficient (KO) CD4^+^CD25^+^ Tregs have reduced ability to inhibit GVHD. (**A**) Lethally irradiated BALB/c mice were given i.v. injections of 5×10^6^ T cell depleted BM cells from B6 mice donors alone (n = 5) or with 1×10^6^ (1 M) or 0.5×10^6^ (0.5 M) splenic naïve T cells (CD25^−^CD62L^+^) from WT (n = 10) or CD73 KO (n = 10) B6 donors. (**B**) Lethally irradiated BALB/c mice were given i.v. injections of 5×10^6^ T cell depleted BM cells from B6 mice donors alone (n = 5) or with 2×10^7^ total splenocytes or CD25 negative (CD25^−^) splenocytes from WT (n = 10) or CD73 KO (n = 10) B6 donors. BM+WT-SP versus BM+CD73 KO-SP, p = 0.0399; BM+WT-SP versus BM+WT-CD25^−^, p = 0.0305. (**C**) Suppression of the alloresponse of B6 CD4^+^CD25^−^ T responder cells (Tres) to BABL/c cells by B6 WT or CD73 KO Tregs (ratio of Tres/Treg; 1∶1) in the MLR. WT Tregs were incubated with or without APCP at 100 µM before co-culture with indicated cells. NECA at 10 µM was added to the co-cultures with CD73 KO Tregs for the duration of the culture. Data are given as means ± SD of triplicates. (**D**) Lethally irradiated BALB/c mice were given i.v. injections of 5×10^6^ T cell depleted BM cells from B6 mice donors alone (n = 5) or plus 5×10^5^ purified B6 WT (n = 10) or CD73 KO (n = 10) CD4^+^CD25^−^ T cells ±5×10^5^ CD4^+^CD25^+^ Tregs. BM+WT-T versus BM+WT-T+Treg, p<0.0001; BM+WT-T+WT-Treg versus BM+WT-T+CD73 KO-Treg, p = 0.024.

To exclude a dose-response effect as the basis for differential GVHD induction, we compared the ability of WT and CD73 KO naive T cells to cause GVHD at two cell doses. At 1 × 10^6^/mouse, both WT and CD73 KO T cells induced 100% GVHD lethality within 42 days after transplantation. As expected, GVHD in recipients of low-dose (0.5 × 10^6^/mouse) WT or CD73 KO T cells was still comparable, but milder than that in high-dose WT T recipients, reflected as recipient survival (Figure 3A). It has been reported that CD73 deficiency reduces function of the CD4^+^CD25^+^ T regulatory (Treg) cells [18], [20] that suppress GVHD development [34], [35]. To assess the role of Treg CD73 in GVHD we removed CD25^+^ cells and compared GVHD induced by CD25^−^ WT or CD73 KO splenocytes. The removal of CD25^+^ cells from WT donor mice significantly accelerated the development of GVHD. Different from total CD73 KO splenocyte transfers (Figure 1B), GVHD lethality of CD25^−^ WT T cell recipients was comparable to that of CD25^−^ CD73 KO T cell recipients, suggesting a role of WT versus CD73 KO CD25^+^ cells in GVHD development (Figure 3B). To dissect the precise role of CD73 by CD4^+^CD25^+^ Tregs in GVHD further, we examined the suppressive activity of C57BL/6 CD4^+^CD25^+^ Tregs in the allogeneic MLR (Figure 3C). CD73 KO Tregs were less able to inhibit the IFN-γ production than WT Tregs, and this effect was abrogated in the presence of 5′-N-ethylcarboxamidoadenosine (NECA, adenosine analogue). Similarly, the inhibitory activity of WT Tregs was blocked by addition of CD73 enzymatic inhibitor APCP, suggesting a requirement of CD73 enzymatic activity in Treg-mediated immune suppression. To assess the in vivo suppression of Tregs to GVHD, we transplanted lethally irradiated recipients with either 5×10^6^ B6 TCD BM or TCD BM plus sorted CD4^+^CD25^−^ T cells (5×10^5^) with or without equal numbers of sorted CD4^+^CD25^+^ Tregs. Mice given TCD BM cells alone appeared healthy, and 100% survived for at least 70 days. WT and CD73 KO CD4^+^CD25^−^ T cells were equally efficient in inducing GVHD and recipient mice died within 32 days after transplantation. In contrast, donor CD73 KO Tregs were less able than WT Tregs to suppress GVHD induced by WT donor CD4^+^CD25^−^ T cells (Figure 3D). Tregs were detected in the spleen and liver of recipients for up to 60 days post transplantation with about 1–3% of all donor CD4^+^ T cells expressing Foxp3. The percentage and absolute of these cells in recipients receiving WT Tregs were similar to those receiving CD73 KO Tregs. Collectively, these data clearly demonstrate that Treg CD73 contributes to protection against GVHD. However, as CD73 KO Tregs also significantly inhibited GVHD, but to a lesser extent than WT Tregs, mechanisms in addition to Treg CD73 also appear to mitigate GVHD.

### Major Role of Recipient CD73 in GVHD Prevention

**Figure 4:**
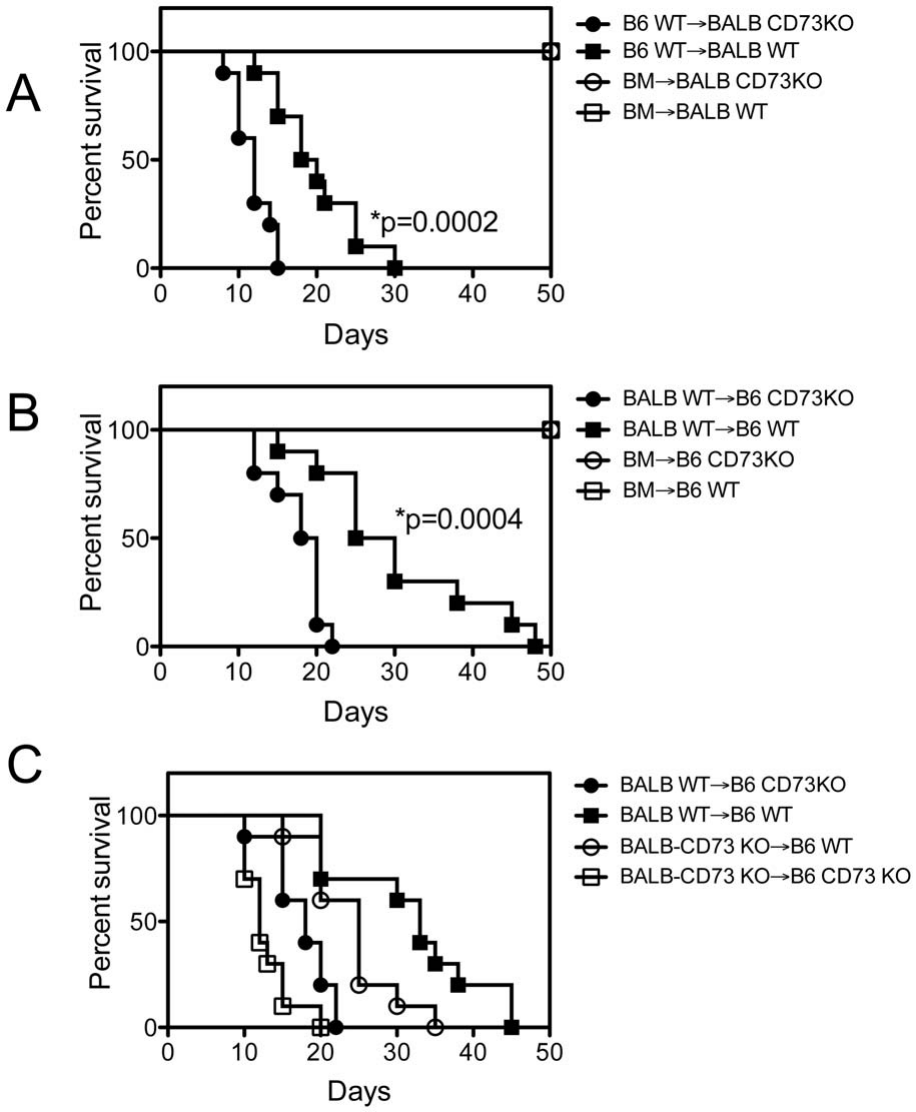
Recipient CD73 limits GVHD development. (**A**) Lethally irradiated WT (n = 10) or CD73 KO (n = 10) BALB/c mice were given i.v. injections of 5×10^6^ T cell depleted BM cells from B6 mice donors alone (n = 5) or with 2×10^7^ splenocytes from WT B6 donors. Lethally irradiated WT (n = 10) or CD73 KO (n = 10) B6 mice were given i.v. injections of 5×10^6^ T cell depleted BM cells from BALB/c mice donors alone (n = 5) or with 2×10^7^ splenocytes from WT BALB/c donors (**B**) or CD73 KO BALB/c donors (**C**). (**A,B**) P values indicate the differences between WT versus CD73 KO recipients. (**C**) BALB WT→B6 CD73KO versus BALB CD73KO→B6 CD73KO, p = 0.0103; BALB WT→B6 CD73KO versus BALB CD73KO→B6 WT, p = 0.0029; BALB CD73KO→B6 WT versus BALB CD73KO→B6 CD73KO, p<0.0001; BALB WT→B6 WT versus BALB CD73KO→B6 WT; p = 0.0284. Results are representative of 2 independently performed experiments with similar results.

Besides lymphocytes, endothelial [31], epithelial [36] and other cells also express CD73. Thus, we examined the contribution of recipient CD73 in acute GVHD in both C57BL/6 → BALB/c (Fig. 4A) and BALB/c → C57BL/6 (Fig. 4B, C) models. Indeed, WT recipient mice survived longer than CD73 KO recipient mice receiving allogeneic cells (Figure 4A, B), suggesting a role of recipient CD73 in GVHD protection. To address further a specific contribution of CD73 on donor versus recipient cells in limiting GVHD, the reciprocal transfers using CD73 KO transfer into WT or CD73 KO recipients were conducted (Figure 4C). In the absence of donor CD73, CD73 KO recipients had shorter survival time than WT recipients, confirming a contribution of recipient CD73 in GVHD (median survival, 12 versus 25 days, p<0.0001). Similarly, in the absence of recipient CD73, recipients receiving WT splenocytes survived longer than those receiving CD73 KO splenocytes, confirming a contribution of donor CD73 in GVHD (median survival, 18 versus 12 days, p = 0.0103), although to a lesser extent. Thus, CD73 deficiency in donors or recipients significantly increased GVHD mortality.

**Figure 5:**
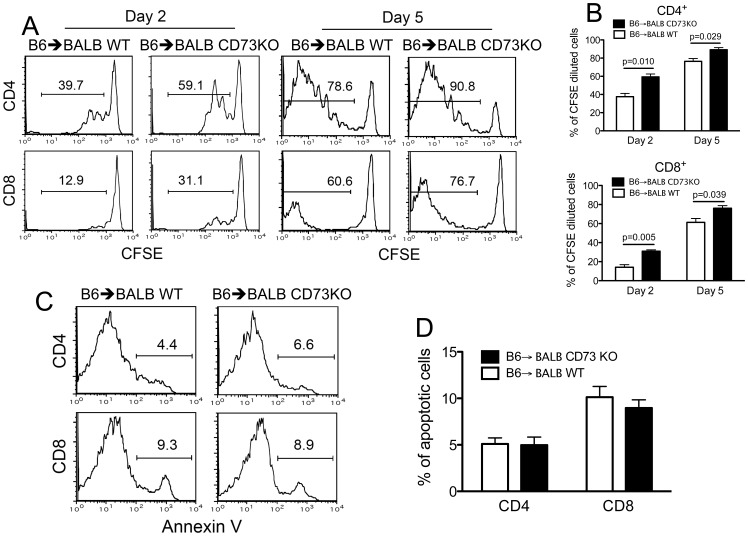
Enhanced proliferation but normal survival of allogeneic donor T cells in CD73 KO recipients. (**A**) Sublethally irradiated WT or CD73 KO BALB/c mice were given i.v. injections of 2×10^7^ dye (CFSE)-labeled splenocytes from WT B6 donors. Division of host-reactive donor T cells was measured 2 and 5 days after cell transfer. CFSE intensity was gated on H-2K^d^-negative (donor) CD4^+^ or CD8^+^ T cells. The percentage of donor T cells with more than one division is indicated in each panel and summarized in (**B**). (**C**) Annexin V staining of donor cells gated on H-2K^d^-negative CD4^+^ or CD8^+^ T cells 5 days after cell transfer. The percentage of apoptotic donor T cells (annexin V^+^) is indicated in each panel (**C**) and summarized in (**D**). Data are given as means ± SEM. Results are representative of 3 independently performed experiments with similar results.

We next investigated the cellular mechanisms of enhanced GVHD lethality in CD73 KO recipients. We hypothesized that recipient CD73 deficiency facilitated alloreactive T-cell activation and/or proliferation that worsened GVHD. To test these possibilities, we first compared the *in vivo* proliferation kinetics of donor T cells. Two to 5 days after transfer, division of donor T cells labeled with carboxyfluorescein succinimidyl ester (CFSE) was examined in WT versus CD73 KO recipients. Both CD4^+^ and CD8^+^ donor T cells in WT recipients divided less than those in CD73 KO recipients (Figure 5A, B), suggesting an important role of recipient CD73 in limiting proliferation of alloreactive T cells. Subsequently, apoptotic cell death in the transferred donor T cells in recipient spleen was evaluated. The percentage of annexin V–positive cells in transferred donor T cells was comparable between WT and CD73 KO recipients (Figure 5C, D). Taken together, our results indicate that lack of recipient CD73 exacerbates GVHD likely by promoting donor T cell proliferation. The mechanism by which recipient CD73 deficiency enhances GVHD could be due to less accumulation of recipient extracellular adenosine that has been demonstrated to inhibit T cell activation and/or proliferation *in vivo* [19], [20].

Because host DCs are critical for the induction of GVHD after irradiation conditioning [37], [38], we also determined if CD73 is required for host DC-initiated GVHD. Given that MHC class II-deficient mice are resistant to CD4^+^ T cell-dependent GVHD [39], we transferred MHC-II expressing DC from C57BL/6 WT or CD73 KO mice in the BALB/c → C57BL/6 II KO GVHD model. As previously reported, compared to WT hosts, MHC class II-deficient hosts were resistant to GVHD mortality. The addition of MHC-II expressing WT DC to MHC class II-deficient recipients significantly increased GVHD mortality, equivalent to the addition of CD73 KO DC (Figure S3A). Moreover, the accumulation of transferred donor alloreactive T cells (Figure S3B) and serum levels of TNF-α (Figure S3C) were elevated to a similar extent by the addition of MHC-II expressing WT DC and CD73 KO DC. These data indicate that CD73 is dispensable on host DC for the induction of GVHD.

### Pharmacological Blockade of CD73 Activity Enhances GVHD Development

**Figure 6:**
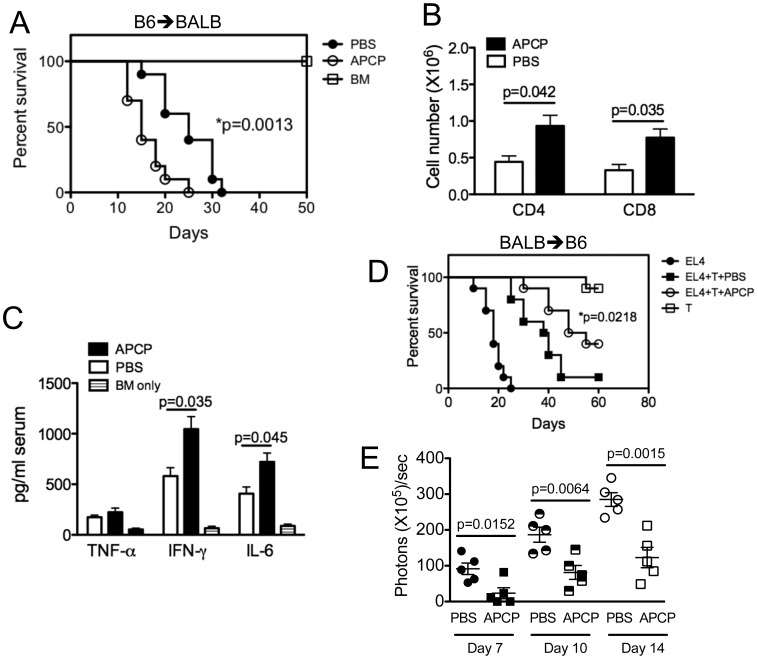
Blockade of CD73 enzymatic activity using the CD73-selective inhibitor APCP exacerbates GVHD but enhances GVL effects. (A) Lethally irradiated BALB/c mice were given i.v. injections of 5×10^6^ T cell depleted BM cells from B6 mice donors alone (n = 5) or with 2×10^7^ splenocytes from WT B6 donors. In the groups receiving T cell transfer, the mice were treated with PBS or APCP 20 mg/kg i.v. twice weekly following donor BM and T cell transfer. (B) Enriched B6 WT T cells were injected i.v. into lethally irradiated BALB/c mice at 2×10^6^ per mouse, and cell expansion was determined 14 days after cell transfer. Mean of absolute number of CD4^+^ or CD8^+^ cells per spleen was shown in recipients given donor (H-2K^b+^) T cells (n = 5). (C) Indicated cytokines were measured in recipient serum on day 14 after donor BM and T cell transfer as described above (n = 5). (D) Lethally irradiated B6 mice (8–10 per group) were given i.v. injections of 5×10^6^ T cell-depleted BM cells from BALB/c mice donors alone or with 10^6^ BALB/c splenic T cells. In the groups receiving T cell transfer, the mice were treated with PBS or APCP 20 mg/kg i.v. twice weekly following donor BM and T cell transfer. Recipient mice were given 10^5^ EL4-luc lymphoma cells as a separate i.v. injection at the same time of transfer. The recipient mice were monitored daily for survival. (E) The average of relative bioluminescence signal intensity of 5 mice per group at different time points as indicated was shown. P values indicate the differences between control PBS versus APCP treatment. Data are given as means ± SEM.

To confirm the effects observed in CD73 KO recipient mice (Figure 5) and/or mice receiving CD73 KO splenocytes (Figure 1), we employed the small molecule inhibitor APCP interfering with CD73 enzymatic activity. Pharmacological inhibition of CD73 activity using APCP has been validated in various murine models by our group [19], [20] and others [22]. Consistent with a role for CD73 in mitigating GVHD, CD73 blockade by APCP administration significantly accelerated GVHD development (Figure 6A). Moreover, APCP treatment increased absolute number of transferred donor T cells in recipient spleen (Figure 6B).

We then asked whether CD73 blockade affects the production of the inflammatory cytokines IL-6, IFN-γ, and TNF-α that are known to contribute to GVHD pathogenesis [4], [5]. On day 14 after transplantation there was more serum IL-6 and IFN-γ following APCP treatment versus PBS treatment (Figure 6C). These data indicate that CD73 enzymatic activity is closely implicated in GVHD development by reducing proinflammatory cytokine production.

CD73 deficiency augments alloreactivity in GVHD models. Thus, we next assessed the effects of CD73 blockade on the GVL activity of alloreactive T cells in the BALB/c → C57BL/6 GVHD model using luciferase transfected-EL4 lymphoma cells (Figure 6D,E). Recipients were monitored for survival daily and for tumor growth using *in vivo* bioluminescent imaging weekly. As expected, recipients of TCD BM plus EL4 cells had rapid death within 25 days after transplantation (Fig. 6D). Most recipients of TCD BM plus WT T cells with EL4 cells died within 45 days after transplantation. By contrast, recipients of TCD BM plus T cells with EL4 cells treated by APCP had a much-delayed death, and a proportion of these recipients had long-term survival (Fig. 6D). In support of these results, CD73 blockade by APCP inhibited EL4 tumor growth *in vivo*, as reflected by quantification of bioluminescent intensity on days 7, 10 and 14 (Fig. 6E). These data indicate that CD73 blockade enhances GVL activity. Because the alloreactive T cell dose used did not significantly contribute to GVHD mortality (Fig. 6D), the death of these recipients was likely due to tumor growth.

### A2AR Signaling is Required for CD73-mediated GVHD Protection

**Figure 7:**
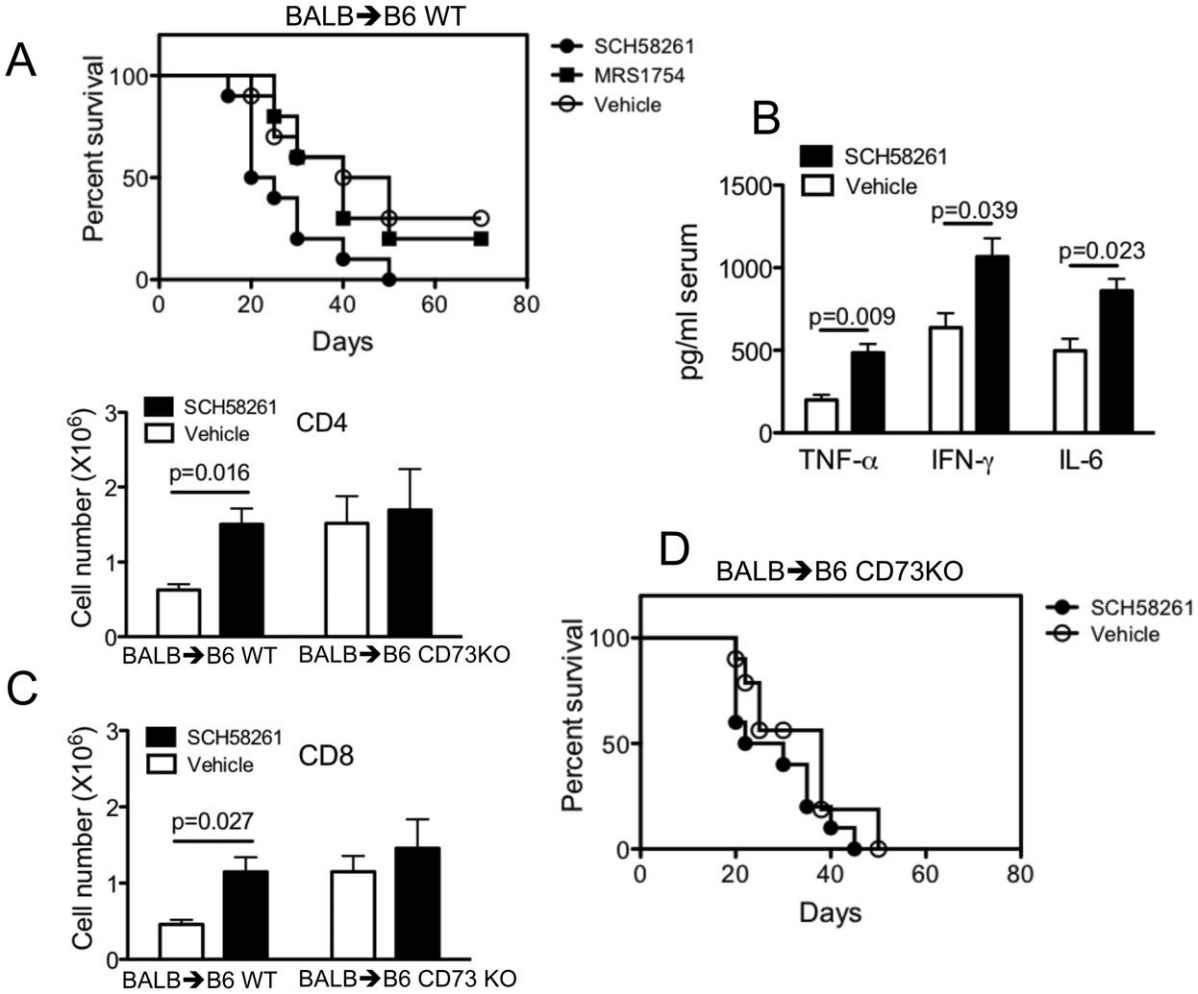
A2AR antagonist administration worsens GVHD. (**A**) Lethally irradiated B6 mice were given i.v. injections of 5×10^6^ T cell depleted BALB/c BM cells with 2×10^7^ splenocytes. Recipients received daily i.p. injections of 2 mg/kg SCH58261, 2 mg/kg MRS1754 or vehicle (0.1% DMSO). Injections were initiated 48 h before cell transfer and continued for 14 days. SCH58261 versus vehicle, p = 0.0204. (**B**) Indicated cytokines were measured in recipient serum on day 14 after donor BM and T cell transfer as described above (n = 5). (**C**) Enriched BALB/c WT T cells were injected i.v. into lethally irradiated B6 WT or CD73 KO mice at 2×10^6^ per mouse, and cell expansion was determined 14 days after cell transfer. Recipients received daily i.p. injections of 2 mg/kg SCH58261 or vehicle (0.1% DMSO) as described above. Mean of absolute numbers of CD4^+^ or CD8^+^ cells per spleen was shown in recipients given donor T cells (n = 5). (**D**) Lethally irradiated B6 CD73 KO mice were given i.v. injections of 5×10^6^ T cell-depleted BALB/c BM cells with 2×10^7^ splenocytes. Recipients received daily i.p. injections of 2 mg/kg SCH58261 or vehicle (0.1% DMSO) as described above.

Adenosine regulation of immune cell function through A2AR and A2BR is an important mechanism to prevent death from inflammation-induced tissue injury/damage. To explore the role of A2AR and A2BR in GVHD, we tested the effect of pharmacological blockade of A2AR or A2BR in the BALB/c → C57BL/6 GVHD model. As shown in Fig. 7A, administration of the A2AR antagonist SCH58261 increased GVHD mortality, as compared with vehicle-treated mice (p = 0.0204). By contrast, overall survival of recipients treated with the A2BR antagonist MRS1754 was not significantly different from vehicle-treated mice, suggesting that A2AR, rather than A2BR primarily contributes to GVHD protection. Indeed, we found that A2AR blockade enhanced serum concentrations of the inflammatory cytokines IL-6, IFN-γ, and TNF-α (Fig. 7B). To determine the role of A2AR further in CD73-mediated GVHD protection, the effect of pharmacological blockade of A2AR was compared between WT and CD73 KO recipients. As expected, SCH58261 administration increased absolute numbers of transferred donor alloreactive T cells in WT recipients but not in CD73 KO recipients (Fig. 7C). Moreover, overall survival of recipients treated with SCH58261 was not significantly different from vehicle treatment when recipients lacked CD73 (Fig. 7D). These results are compatible with the concept that the proliferation of allogeneic T cells is inhibited by signaling through A2AR by endogenous CD73-generated adenosine. Together, our data indicate an important role for CD73 in limiting GVHD by the production of extracellular adenosine for A2AR activation.

## Discussion

Although a potential role for CD73-generated endogenous adenosine in pathogenesis of various solid allograft models has been reported [11], [27], [28], definite evidence for the direct involvement of CD73 in GVHD pathogenesis has not emerged. In the present study, we used CD73 KO mice as donors or recipients in GVHD models to demonstrate an essential requirement for CD73 in alloreactivity and GVHD. This finding is consistent with recent studies [40], but adds more unique aspects to the potential contribution of CD73 on donor cells (e.g. Treg) or host cells (e.g. DC) to control the severity of GVHD. This requirement could be attributed to the phosphohydrolysis of AMP by CD73 because APCP, a well-established inhibitor of the enzyme activity of CD73, had been tested and confirmed for this purpose in GVHD. However, there are also likely functions of CD73 in GVHD that are independent of its enzymatic activities. Other CD73 activities in different regards include structural interaction [41], integrin-mediated binding [42], [43] and intracellular signaling [6]. These possible mechanisms need further investigation.

Using two distinct mouse models of alloreactivity (parent-to-F1 and fully MHC-mismatched), We first demonstrated that donor cell CD73 status is implicated in GVHD immunopathogenesis. To dissect donor CD73 contributions to GVHD pathogenesis further, we focused on two major donor T cell populations: CD3^+^CD25^−^CD62L^+^ naïve T cells and CD4^+^CD25^+^ Treg that both express CD73. Since acute GVHD is primarily a T-cell-mediated disease, it is likely that enhanced alloreactivity of CD73 KO donor T cells exacerbates GVHD development. However, we found that CD73 deficiency did not affect naïve T cell activation or proliferation in response to alloantigen *in vitro* or *in vivo*. In agreement, our previous work showed that CD4^+^ and CD8^+^ CD73 KO or WT T cells proliferated equally in response to antigen-independent T cell receptor activation *in vitro,* and there was no significant difference in *in vivo* proliferation or IFN-γ production between CD73 KO and WT antigen-specific T cells in a tumor model [20]. A recent *in vitro* study reported that CD73-derived adenosine suppressed NF-κB activation in CD4^+^ T cells, thereby regulating the release of an array of proinflammatory cytokines and chemokines [44]. We do not know whether this CD73-mediated repression of active NF-κB in T cells occurs *in vivo* particularly in a setting of GVHD. Importantly, we showed that both CD3^+^CD25^−^CD62L^+^ naïve T cells and CD4^+^CD25^−^ T cells from CD73 KO donor mice were nearly equivalent in inducing GVHD lethality compared to WT donor counterparts. Thus, it is most likely that donor naïve T cell CD73 plays a negligible role in GVHD development.

Accumulating animal model and clinical evidence has demonstrated the requirement for Tregs in transplantation tolerance [45]. The regulatory properties of Tregs make them ideal for therapeutic applications in transplantation. For example, polyclonal Tregs were efficient at controlling undesired immune responses in mouse models of GVHD [34], [35]. The importance of CD39 and CD73 in the functional activity of Treg through the production of adenosine has been recently highlighted in transplantation [46]. For example, in a murine adoptive cell transfer model, Tregs from CD39 KO mice were less efficient in preventing rejection of allogeneic skin grafts than WT Tregs expressing CD39 [11]. Likewise, adenosine production by adoptively transferred CD73 KO Tregs failed to prevent gastritis as efficiently as transferred WT Tregs [18]. Moreover, we previously showed that CD73-generated adenosine by Tregs inhibited anti-tumor T cell immunity and facilitated tumor growth [20]. We then considered Tregs as a possible CD73-expressing defense against GVHD. We established that donor CD73 KO Tregs were less able than WT Tregs to suppress GVHD development, suggesting that Treg CD73 helps mitigate GVHD immunopathology.

Donor cells may not be the only effector cells relevant to GVHD that are affected by CD73. In fact, recipient CD73 plays a major role in determining the severity of GVHD. It is possible that recipient CD73 on BM-derived cell populations, such as certain subsets of B cells and DCs [47]–[50], contributes to GVHD. Indeed, it has been reported that host BM-derived DC through conditioning are critical for the induction of GVHD [37], [38]. However, our data show that alloreactive T cell responses induced by WT or CD73 KO DCs are comparable, and CD73 is dispensable on recipient DC for the induction of GVHD, suggesting a primary role of non-BM-derived recipient CD73 in GVHD, although a potential contribution from other recipient BM cells is not entirely excluded. Thus, we need to further clarify which non-hematopoietic cells of the recipient express CD73 (e.g. endothelial cells, epithelial cells, enterocytes, fibroblasts etc.). Non-BM-derived cells such as endothelial cells [31] and epithelial cells [36] can express CD73. Recent studies have demonstrated that activated allogeneic T cells traffic through the circulation, secondary lymphoid organs to parenchymal target organs such as gastrointestinal tract, liver, skin, and lung, where they induce tissue damage during development of GVHD [2], [51]. Thus, it is possible that the immunological crosstalk between endothelial/epithelial and T cells through CD73 activity is involved in GVHD development. In accordance with this hypothesis, an elegant study reported that CD73-dependent adenosine generation regulates allogeneic interactions between endothelial cells and T lymphocytes and thus plays an immunomodulatory role that promotes cardiac allograft survival [27]. Another recent study has shown that loss of CD73 in tracheal transplant recipients resulted in an exacerbated immune response toward the allograft manifested by increased T cell infiltration, proinflammatory cytokine production and graft luminal occlusion [28]. Furthermore, CD73 influences ion and fluid transport in a variety of mucosal epithelial cell types [7]. At present, it is not known how recipient CD73 directly influences the pathogenesis of GVHD aside from the Treg and proinflammatory cytokine contributions that we now report. Further work is thus needed to understand the role of recipient CD73 on endothelial cells or epithelial cells in GVHD.

There are four adenosine receptors: A1, A2A, A2B, and A3. Signaling through A2AR in CD73-mediated attenuation of allograft airway rejection [28], and through A2BR in cardiac allograft vasculopathy [27] have previously been demonstrated. Moreover, activation of A2AR using the selective agonist ATL146e limits GVHD after allogeneic hematopoietic stem cell transplantation [52]. In line with these results, we used selective A2AR and A2BR antagonists to demonstrate an important role for CD73 in limiting GVHD by the production of extracellular adenosine through A2AR activation rather than A2BR activation.

Dissociation of GVHD and GVL remains of paramount importance in improving the efficacy of bone marrow transplantation. An idea goal for GVHD prevention and therapy is to inhibit T cell activities that mediate GVHD while preserving the benefit of T cell–mediated GVL. Since GVHD induction typically associates with GVL effects, the mechanisms by which T cells specifically retain GVL activity are presently unclear. Pharmacological blockade of CD73 by the selective inhibitor APCP or anti-CD73 monoclonal antibodies (mAb) has the potential to provide numerous anti-tumor effects [19]–[21], both direct and indirect. Considering that leukemia cells are better targets for alloreactive T cells than epithelial cells (which are targeted in GVHD), the ability of CD73 blockade to mitigate GVHD and simultaneously prevent GVL loss rests on achieving an appropriate balance between these effects. Indeed, we found that CD73 blockade augments GVL activity while the alloreactive T cell dose used did not significantly contribute to GVHD mortality. Therefore, either enhancing or blocking CD73 activity has great potential clinical application in allogeneic transplants.

In summary, our data demonstrate the importance of CD73-generated endogenous adenosine for protection from GVHD development. Both donor and recipient CD73 are protective in GVHD immunopathogenesis. Treg CD73 specifically helps defend against GVHD, although CD73 on non-hematopoietic cells also plays a role that is incompletely defined at present. These findings clarify the role of adenosine metabolism in the immunopathogenesis of GVHD and provide a sound rationale for boosting the CD73-mediated adenosinergic effects to treat GVHD. Inhibiting CD73 for GVL appears challenging, and further work is required to determine if an appropriate balance between GVHD and GVL through manipulation of the CD73/adenosine axis is clinically possible.

## Materials and Methods

### Mice and Reagents

WT C57BL/6 (B6, H-2^b^), BALB/c (H-2^d^) and B6D2F1 (B6 X DBA/2; H-2^bXd^) hybrid mice were purchased from NCI-Frederick. MHC class II deficient mice (B6.129S-H2dlAb1) were purchased from The Jackson Laboratory (Bar Harbor, ME). CD73^−/−^ mice (CD73 KO) were generated as described previously [29], and back-crossed on to the C57BL/6 (H-2^b^) and BALB/c (H-2^d^) backgrounds for 14 generations. Age- and sex-matched 8- to 10-week-old mice were used for all experiments. All animal experiments were approved by the institutional animal care and use committee of the University of Texas Health Science Center at San Antonio. All the mAbs were obtained from eBioscience and BioLegend. The Alexa Fluor 647 Annexin V apoptosis detection kit and ELISA kit were from BioLegend. The CD73 selective inhibitor α,β-methylene adenosine 5′-diphosphate (APCP) and SCH58261 (A2AR antagonist) were purchased from Sigma. MRS 1754 (A2BR antagonist) and 5′-N-ethylcarboxamidoadenosine (NECA) were from Tocris Bioscience.

### T Cell Purification, DC Isolation and Harvesting Bone Marrow Cells from donor Mice

T cells were purified from pooled spleen cells by negative selection through the Pan T Cell Isolation Kit (Miltenyi Biotech). Naïve T cells were further purified to remove CD25^+^ cells by anti-CD25 MicroBeads (Miltenyi Biotech). Periodic flow cytometric analysis routinely showed >90% purity of CD25^−^CD62L^+^ cells. CD4^+^ CD25^+^ T cells (Tregs) were sorted from pooled spleen cells by BD FACSAria flow sorter with greater than 95% purity. To enrich splenic DCs, mice were injected s.c. once daily with 10 µg of recombinant human flt3 ligand (eBioscience) for 8 consecutive days. CD11c^+^ splenic DCs were subsequently isolated by using anti-CD11c MicroBeads (Miltenyi Biotech) from spleens treated with Librase Blendzyme II (Roche Diagnostics). BM was harvested from tibia and femurs, and T cells were depleted through anti-Thy1.2 MicroBeads (Miltenyi Biotech).

### Analysis of Cells by Flow Cytometry

All samples were initially incubated with 2.4G2 to block antibody binding to Fc receptors. Single cell suspensions were stained with 1 µg of relevant fluorophore-conjugated mAbs or isotype controls and then washed twice with cold PBS. Samples were analyzed on a LSRII and data were analyzed with FlowJo software.

### Mouse GVHD Models

For BM transplantation model with lethal irradiation, mice were conditioned with 10 Gy of total body irradiation from ^137^Cs source at 110 cGy/minute. Within 24 hours after irradiation, the irradiated mice received the T cell–depleted BM cells with or without splenocytes or T cells by i.v. tail vein injection using a restrainer. Sulfamethoxazole trimethoprim (0.2 mg/ml) was added to the drinking water of those irradiated mice for preventing bacterial infections starting at the day before irradiation and through out the entire experiments. Mice were monitored daily in the first 2 weeks and every other day afterwards for clinical signs of GVHD, such as ruffled fur, hunched back, inactivity or diarrhea, and mortality. Animals judged to be moribund were euthanatized and counted as GVHD lethality.

The mouse models of GVHD investigated are described in Table S1. In the fully MHC–mismatched GVHD model, WT or CD73 KO BALB/c mice were exposed to lethal irradiation (10 Gy) followed by intravenous (i.v.) transfer of TCD B6 BM cells (5×10^6^ cells) with or without B6 T cells or splenocytes isolated from WT, or CD73 KO mice. Sections of lung, liver, skin and colon tissues collected 10 days after BM transplantation (BMT) were stained with hematoxylin/eosin (H&E) and scored by two trained pathologists blinded to the treatment groups as described previously [53]. Cohorts of WT B6 splenocyte recipients were administered with PBS or APCP 20 mg/kg i.v. twice weekly following donor BMT. To study the role of CD73 on Tregs in limiting GVHD, BALB/c mice were given i.v. injections of 5×10^6^ T cell-depleted BM cells from B6 mice donors alone or plus 5×10^5^ purified B6 CD73 KO or WT CD4^+^CD25^−^ T cells ±5×10^5^ CD4^+^CD25^+^ Tregs from B6 WT or CD73 KO mice. On the other hand, B6 WT or CD73 KO mice as recipients were exposed to lethal irradiation (10 Gy) followed by i.v. transfer of T cell–depleted BALB/c BM cells (5×10^6^ cells) with or without BALB/c splenocytes. Cohorts of WT B6 recipients were injected daily i.p. with 2 mg/kg SCH58261, 2 mg/kg MRS1754 or vehicle (0.1% DMSO). Injections were initiated 48 h before cell transfer and continued for 14 days. To study if CD73 is required for host DC-initiated GVHD, lethally irradiated B6 WT and MHC-II deficient mice were given i.v. injections of 5×10^6^ T cell depleted BALB/c BM cells with 5×10^6^ splenic CD4^+^ T cells. Cohorts of MHC-II deficient mice were injected with 5×10^6^ B6 WT or CD73 KO DCs following irradiation. The recipient mice were monitored daily for survival and every 5 days for body weight changes.

In the parent-to-F1 GVHD model, B6D2F1 recipient mice, were lethally irradiated (10 Gy), and injected i.v. with T cell–depleted B6 BM cells (5×10^6^ cells) with or without B6 splenocytes (2–3×10^7^ cells) isolated from either WT or CD73 KO mice. The recipient mice were monitored daily for survival and every 5 days for body weight changes. Blood samples were obtained from BMT recipients at times specified, and IFN-γ, TNF-α and IL-6 were measured in recipient serum by ELISA.

### Mouse GVL Model

In GVL studies, lethally irradiated B6 mice were given i.v. injections of 5×10^6^ T cell-depleted BM cells from BALB/c mice donors alone or with 10^6^ BALB/c splenic T cells. In the groups receiving T cell transfer, the mice were treated with PBS or APCP 20 mg/kg i.v. twice weekly following BMT. To establish a leukemia/lymphoma in the BMT recipients, 10^5^ mouse luciferase-expressing EL4 cells (T cell lymphoma line derived from B6 mice, EL4-luc) were given on the day of BMT. All experiments were conducted with 8–10 mice per group. To distinguish mortality due to GVHD or tumor relapse, we used bioluminescence imaging to monitor tumor growth *in vivo*. Mice that received EL4-luc were given i.p. (150 mg/kg) D-Luciferin (Xenogen, Alameda, CA), and luciferase activity was measured 10 minutes after injection in a Xenogen IVIS bioluminescence imaging system. The signal from EL4-luc cells was quantified in photons/s/mouse.

### Measurement of in vivo Proliferation and Apoptosis of Donor Cells

Splenocytes were labeled with CFSE (Invitrogen, Carlsbad, CA) and transferred into irradiated allogeneic recipients. CFSE dilution of H-2K^d^-negative, CD4^+^ or CD8^+^ T cells was analyzed by flow cytometry as described previously [51]. Apoptosis of donor T cells was examined by annexin V staining of H-2K^d^-negative, CD4^+^ or CD8^+^ T cells.

### Measurement of in vivo Th1, Th2 or Th17 Commitment of Donor T Cells

Lethally irradiated B6 mice were given i.v. injections of 1.2×10^7^ T cell depleted BM cells from BALB/c mice WT donors with 3×10^6^ splenic naïve T cells (CD25^−^CD62L^+^) from WT or CD73 KO BALB/c donors. After 5 days, recipient spleens were harvested and the percentages of IFN-γ^+^ (Th1), IL-4^+^ (Th2), or IL-17^+^ (Th17) cells in donor (H-2K^d^ positive) CD4^+^ T cells were determined by flow cytometry.

### Allogeneic Mixed Lymphocyte Reaction (MLR)

T cells were purified from spleen cells by negative selection using the Pan T Cell Isolation Kit (Miltenyi Biotech, Auburn, CA). Splenic CD11c^+^ DCs were sorted on a BD FACSAria Cell sorter. B6 WT or CD73 KO T cells (5×10^5^/well) were co-cultured with 1×10^5^ BALB/c mouse CD11c^+^ DCs for 3 days. To determine the role of CD73 on DCs, BALB/c WT T cells (5×10^5^/well) were co-cultured with 1×10^5^ B6 WT or CD73 KO CD11c^+^ DCs for 3 days. Alloreactive T cell proliferation was measured by ^3^H thymidine incorporation. To measure the suppression of the alloresponse of B6 CD4^+^CD25^−^ T responder cells (Tres) to BABL/c cells by Tregs, B6 CD4^+^CD25^−^ Tres cells (1×10^5^/well) were co-cultured with 3×10^4^ BALB/c mouse CD11c^+^ DCs with or without 1×10^5^ B6 WT or CD73 KO CD4^+^CD25^+^ Tregs in the presence of 100 µM 5′-AMP for 3 days. WT Tregs will be incubated with or without APCP at 100 µM before co-culture with Tres cells. NECA at 10 µM was added to co-cultures as indicated for the duration of the culture. Culture supernatants were collected for IL-2 or IFN-γ measurement by ELISA.

### Statistical Analysis

Kaplan-Meier survival curves were generated using Prism (Graphpad Software, Inc.). Differences in survival of groups of mice were calculated according to the log-rank (Mantel-Cox) test. The statistical significance of other measurements in different groups was determined by Student’s *t* test. Probability values >0.05 were considered non-significant.

## Supporting Information

Figure S1 **The expression patterns of CD44 and CD62L in spleen.** CD62L versus CD44 expression is shown gated on CD4 and CD8 populations in spleen from B6 WT or CD73 KO mice. Results are representative of 3 independently performed experiments with similar results.
(PDF)

Figure S2 **CD73 deficiency has no effect on Th1, Th2 or Th17 commitment by intracellular cytokine staining and gating on donor type T cells.** Lethally irradiated B6 mice were given i.v. injections of T cell depleted BM cells from BALB/c mice donors with splenic naïve T cells (CD25^−^CD62L^+^) from WT or CD73 KO BALB/c donors. After 5 days, recipient spleens were harvested and the percentages of IFN-γ^+^, IL-4^+^, or IL-17^+^ cells in donor (H-2K^d^ positive) CD4^+^ T cells were determined by flow cytometry (n = 3). Numbers in flow panels indicate percent positive cells in each.
(PDF)

Figure S3 **CD73 is not required for host-derived DC-initiated GVHD. (A)** Lethally irradiated B6 WT and MHC-II KO mice (n = 8–10 per group) were given i.v. injections of 5×10^6^ T cell depleted BALB/c BM cells with 5×10^6^ splenic CD4^+^ T cells. Cohort of MHC-II KO mice were injected with 5×10^6^ B6 WT or CD73 KO DCs following irradiation. **(B)** Mean of absolute number of donor T cells per spleen was shown in recipients (n = 5). **(C)** TNF was measured in recipient serum on day 14 after donor BM and T cell transfer as described above (n = 5).
(PDF)

Table S1 **Mouse models of graft-versus-host disease investigated.**
(PDF)
