# Supplementary material for: Graft-versus-Host Disease Is Enhanced by Selective CD73 Blockade in Mice
Source: PLoS One. 2013 Mar 8;8(3):e58397. doi: 10.1371/journal.pone.0058397 (PMC3592842; doi:10.1371/journal.pone.0058397)
Supplement: Figure S1 — The expression patterns of CD44 and CD62L in spleen. CD62L versus CD44 expression is shown gated on CD4 and CD8 populations in spleen from B6 WT or CD73 KO mice. Results are representative of 3 independently performed experiments with similar results. (PDF) [file pone.0058397.s001.pdf]

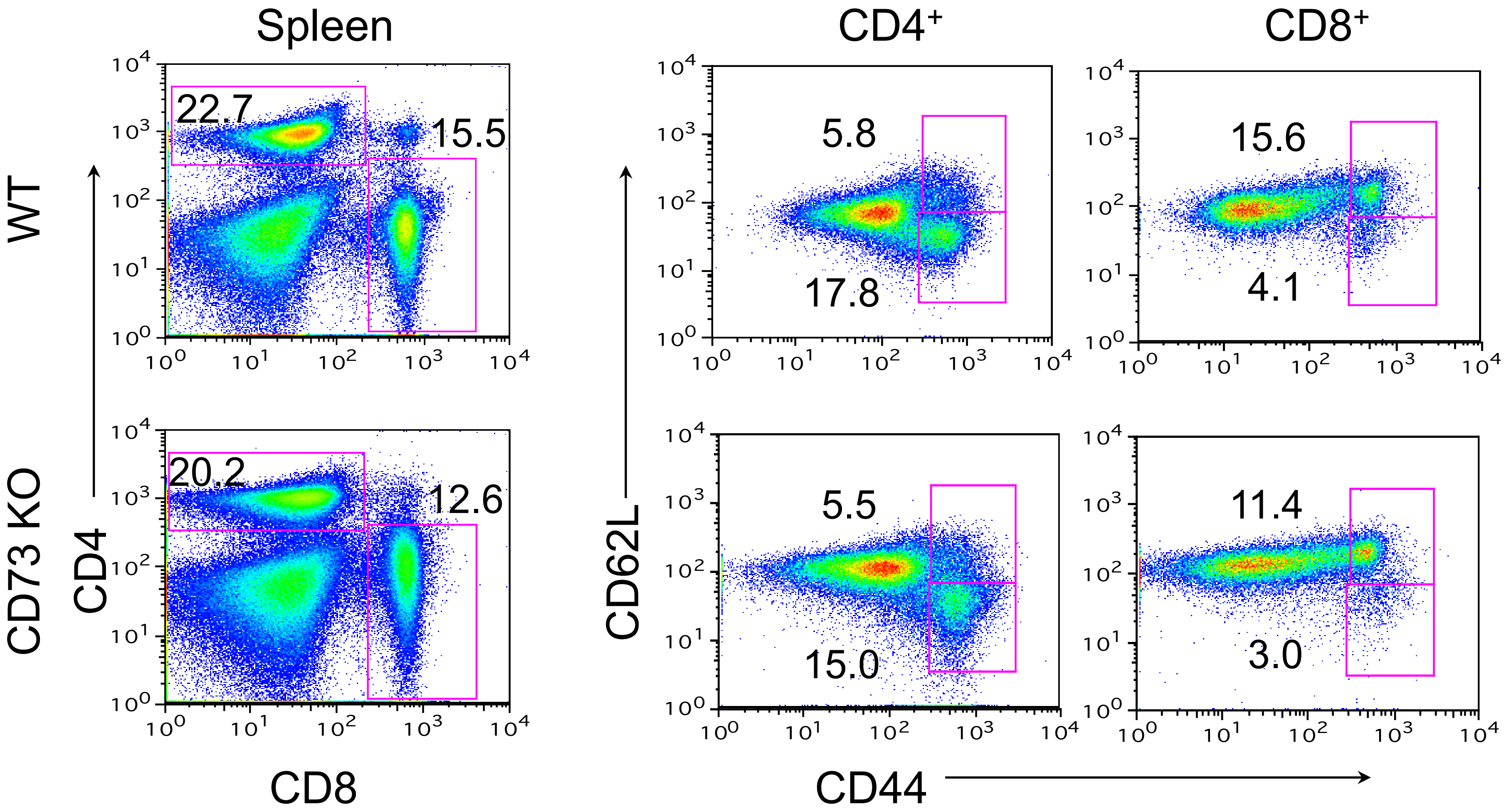

Figure S1. The expression patterns of CD44 and CD62L in spleen. CD62L versus CD44 expression is shown gated on CD4 and CD8 populations in spleen from B6 WT or CD73 KO mice. Results are representative of 3 independently performed experiments with similar results.
