# Supplementary material for: Graft-versus-Host Disease Is Enhanced by Selective CD73 Blockade in Mice
Source: PLoS One. 2013 Mar 8;8(3):e58397. doi: 10.1371/journal.pone.0058397 (PMC3592842; doi:10.1371/journal.pone.0058397)
Supplement: Figure S2 — CD73 deficiency has no effect on Th1, Th2 or Th17 commitment by intracellular cytokine staining and gating on donor type T cells. Lethally irradiated B6 mice were given i.v. injections of T cell depleted BM cells from BALB/c mice donors with splenic naïve T cells (CD25−CD62L+) from WT or CD73 KO BALB/c donors. After 5 days, recipient spleens were harvested and the percentages of IFN-γ+, IL-4+, or IL-17+ cells in donor (H-2Kd positive) CD4+ T cells were determined by flow cytometry (n = 3). Numbers in flow panels indicate percent positive cells in each. (PDF) [file pone.0058397.s002.pdf]

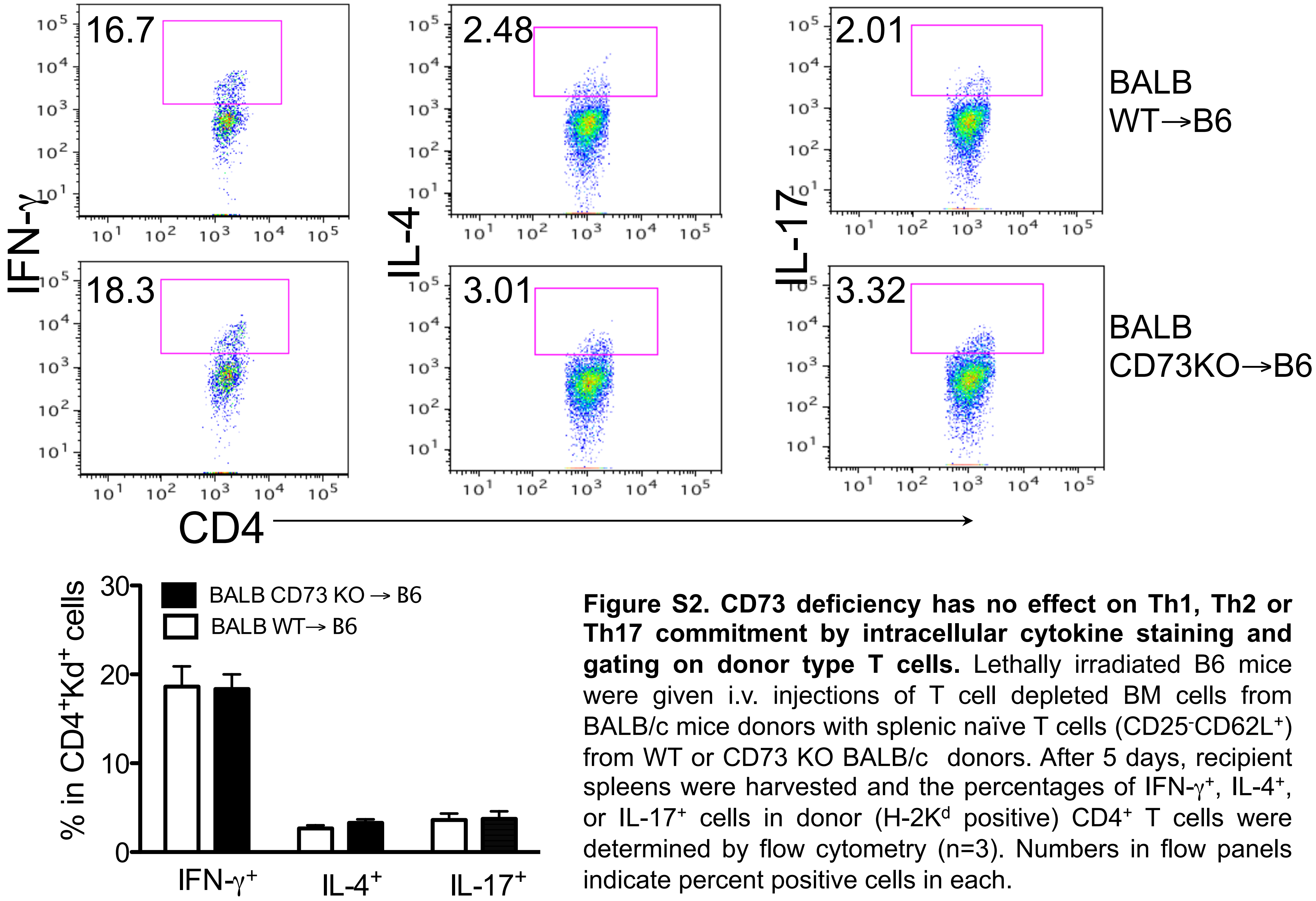


**Figure S2. CD73 deficiency has no effect on Th1, Th2 or Th17 commitment by intracellular cytokine staining and gating on donor type T cells.** Lethally irradiated B6 mice were given i.v. injections of T cell depleted BM cells from BALB/c mice donors with splenic naïve T cells ($CD25^{-}CD62L^{+}$) from WT or CD73 KO BALB/c donors. After 5 days, recipient spleens were harvested and the percentages of IFN-$\gamma^{+}$, IL-$4^{+}$, or IL-$17^{+}$ cells in donor (H-$2K^{d}$ positive) $CD4^{+}$ T cells were determined by flow cytometry (n=3). Numbers in flow panels indicate percent positive cells in each.
