# Supplementary material for: Graft-versus-Host Disease Is Enhanced by Selective CD73 Blockade in Mice
Source: PLoS One. 2013 Mar 8;8(3):e58397. doi: 10.1371/journal.pone.0058397 (PMC3592842; doi:10.1371/journal.pone.0058397)
Supplement: Figure S3 — CD73 is not required for host-derived DC-initiated GVHD. (A) Lethally irradiated B6 WT and MHC-II KO mice (n = 8–10 per group) were given i.v. injections of 5×10^6^ T cell depleted BALB/c BM cells with 5×10^6^ splenic CD4+ T cells. Cohort of MHC-II KO mice were injected with 5×10^6^ B6 WT or CD73 KO DCs following irradiation. (B) Mean of absolute number of donor T cells per spleen was shown in recipients (n = 5). (C) TNF was measured in recipient serum on day 14 after donor BM and T cell transfer as described above (n = 5). (PDF) [file pone.0058397.s003.pdf]

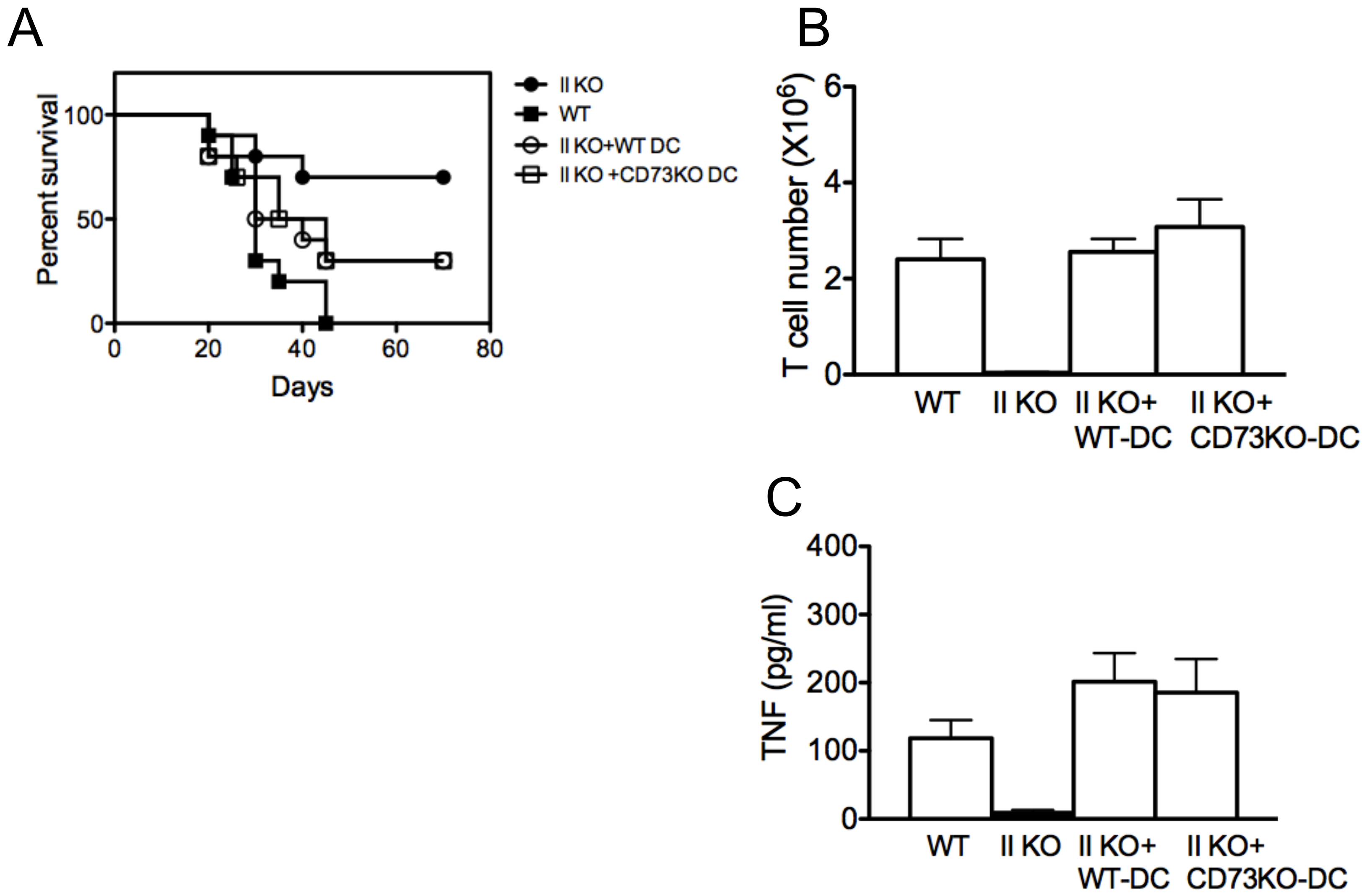

Figure S3. CD73 is not required for host-derived DC-initiated GVHD. (A) Lethally irradiated B6 WT and MHC-II KO mice (n=8-10 per group) were given i.v. injections of 5 x 10^6 T cell depleted BALB/c BM cells with 5 x 10^6 splenic $CD4^+$ T cells. Cohort of MHC-II KO mice were injected with 5 x 10^6 B6 WT or CD73 KO DCs following irradiation. **(B)** Mean of absolute number of donor T cells per spleen was shown in recipients (n=5). **(C)** TNF was measured in recipient serum on day 14 after donor BM and T cell transfer as described above (n=5).
