# Supplementary material for: Graft-versus-Host Disease Is Enhanced by Selective CD73 Blockade in Mice
Source: PLoS One. 2013 Mar 8;8(3):e58397. doi: 10.1371/journal.pone.0058397 (PMC3592842; doi:10.1371/journal.pone.0058397)
Supplement: Table S1 — Mouse models of graft-versus-host disease investigated. (PDF) [file pone.0058397.s004.pdf]

Table S1. Mouse models of graft-versus-host disease investigated

Donor	Recipient	Condi-tioning	Genetics	T-cell contributing phenotype	Cell type for transplantation	Treatment	Figure
C57BL/6($H2^b$)<br>WT<br>CD73 KO<br>CD73 KO<br>WT	BALB/c($H2^d$)<br>WT<br>CD73 KO<br>WT<br>CD73KO	10 Gy	Mismatched for MHC I, MHC II and miHAs	$CD4^+$ or $CD8^+$	TCD BM cells and spleen cells		1A, 1C<br>4A
C57BL/6($H2^b$)	BALB/c ($H2^d$)				TCD BM and $CD25^-$ spleen cells from<br>WT<br>CD73 KO		3B
C57BL/6($H2^b$)	BALB/c ($H2^d$)				TCD BM and WT splenic T<br>+WT Treg<br>+CD73 KO Treg		3A<br>3D
C57BL/6($H2^b$)	BALB/c ($H2^d$)				TCD BM cells and splenic T	PBS<br>APCP	6A
BALB/c ($H2^d$)<br>WT<br>CD73 KO<br>CD73 KO<br>WT	C57BL/6 ($H2^b$)<br>WT<br>CD73 KO<br>WT<br>CD73KO				TCD BM cells and spleen cells or naïve T cells		4B, 4C<br>S2
WT<br>WT	WT<br>MHCIIKO				TCD BM cells and splenic T<br>+WT DC<br>+CD73 KO DC		S3
BALB/c ($H2^d$)	C57BL/6 ($H2^b$)				TCD BM cells and spleen cells	Vehicle<br>SCH58261<br>MRS1754	7A, 7D
C57BL/6($H2^b$)<br>WT<br>CD73KO	B6D2F1($H2^{b/d}$)<br>WT<br>WT				TCD BM cells and spleen cells or naïve T cells		1D, 2A, 2B

TCD BM, T cell-depleted bone marrow cells; miHAs, minor histocompatibility antigens; DC; dendritic cells; SCH58261 (A2AR antagonist), MRS1754 (A2BR antagonist); APCP (CD73 selective inhibitor)
